# Comparison of the gastric microbiome in Billroth I and Roux-en-Y reconstructions after distal gastrectomy

**DOI:** 10.1038/s41598-022-14886-4

**Published:** 2022-06-22

**Authors:** Yoshiro Imai, Sang-Woong Lee, Shoichi Sakaguchi, Nahoko Kato-Kogoe, Kohei Taniguchi, Michi Omori, Ryo Tanaka, Kotaro Honda, Wataru Osumi, Takashi Nakano, Takaaki Ueno, Kazuhisa Uchiyama

**Affiliations:** 1 Department of General and Gastroenterological Surgery, Faculty of Medicine, Osaka Medical and Pharmaceutical University, 2-7 Daigaku-machi, Takatsuki, Osaka 569-8686 Japan; 2 Department of Microbiology and Infection Control, Faculty of Medicine, Osaka Medical and Pharmaceutical University, 2-7 Daigaku-machi, Takatsuki, Osaka 569-8686 Japan; 3 Department of Dentistry and Oral Surgery, Faculty of Medicine, Osaka Medical and Pharmaceutical University, 2-7 Daigaku-machi, Takatsuki, Osaka 569-8686 Japan; 4 Translational Research Program, Faculty of Medicine, Osaka Medical and Pharmaceutical University, 2-7 Daigaku-machi, Takatsuki, Osaka 569-8686 Japan

**Keywords:** Gastric cancer, Microbiome, Surgical oncology

## Abstract

The changes in gastric microbiota following reconstruction after gastrectomy have not been reported. This study aimed to compare the gastric microbiota following Billroth I and Roux-en-Y reconstructions after distal gastrectomy. We enrolled 71 gastrectomized patients with gastric cancer; 31 and 40 underwent Billroth I and Roux-en-Y reconstructions, respectively. During upper gastrointestinal endoscopy, gastric fluid was collected immediately before and 6 months after distal gastrectomy. Deoxyribonucleic acid isolated from each sample was evaluated using 16S ribosomal ribonucleic acid metagenomic analysis. Analysis revealed that the gastric microbiota’s species richness (expressed as the alpha diversity) was significantly lower after than before distal gastrectomy (operational taxonomic units, *p* = 0.001; Shannon index, *p* = 0.03). The interindividual diversity (beta diversity) was significantly different before and after distal gastrectomy (unweighted UniFrac distances, *p* = 0.04; weighted UniFrac distances, *p* = 0.001; Bray–Curtis, *p* = 0.001). Alpha and beta diversity were not significantly different between Billroth I and Roux-en-Y reconstructions (observed operational taxonomic units, *p* = 0.58; Shannon index, *p* = 0.95; unweighted UniFrac distances, *p* = 0.65; weighted UniFrac distances, *p* = 0.67; Bray–Curtis, *p* = 0.63). Our study demonstrated significant differences in gastric microbiota diversity, composition, and community before and after distal gastrectomy but no difference between Billroth I and Roux-en-Y reconstruction after distal gastrectomy.

## Introduction

For many years, the stomach was believed to be a bacteria-free organ because of its high acidity. However, this concept was refuted by the discovery of *Helicobacter pylori* (*H. pylori*) in 1982, followed by the identification of additional stomach microbiota^1,2^. In recent years, the application of bacterial 16S ribosomal ribonucleic acid (16S rRNA) gene sequencing technology for human gut microbiota research has gradually facilitated the comprehensive elucidation of the gastric microbiota^3^. Gastric mucosa and gastric fluid have been reported to contain 102–104 colony-forming units per g or mL^4^.

The gastric microbiota plays an essential role in maintaining gastric homeostasis and has been suggested to be associated with gastric diseases. Studies have demonstrated that the diversity of the gastric microbiota changes with the progression of gastritis, intestinal metaplasia, and gastric cancer^5,6^, and fluctuations in the gastric environment may alter the gastric microbiota. Although changes in the gastric environment after subtotal gastrectomy leads to altered gastric microbiota composition^7^, their effect on the remnant stomach has not been extensively studied.

**Figure 1:**
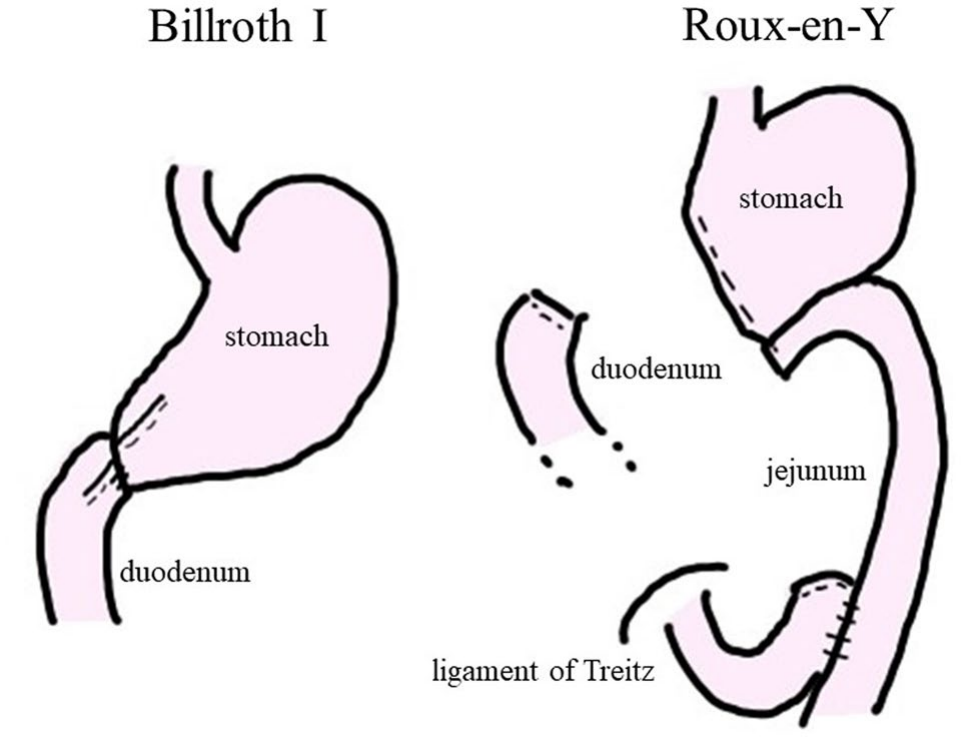
Figure depicting the Billroth I (BI) and Roux-en-Y (RY) reconstructions procedures.

Gastric cancer is the third leading cause of cancer-related deaths worldwide and is the sixth most common cancer globally^8^. Distal gastrectomy (DG) is the most common surgical treatment for gastric cancer, and Billroth I (BI) or Roux-en-Y (RY) reconstructions are commonly performed as standard procedures in Japan (Fig. 1). BI reconstruction following DG is considered simple and relatively easy to perform^9^, and the physiological passage of food and hormone secretion are excellent. However, bile regurgitation leads to remnant gastritis and impaired symptoms. RY reconstruction following DG is a rather complicated procedure; however, it is considered superior to BI reconstruction in terms of bile reflux^10^.

In addition, it is suspected that there is a difference in the gastric environment between BI and RY reconstruction following DG due to bile regurgitation. Although the gastric microbiota is altered after gastrectomy, it is unclear whether and how it differs based on the reconstructive method utilized following DG. Therefore, this study aimed to examine the microbiota in the remnant stomach in BI and RY reconstructions conducted after DG by analyzing gastric fluid using 16S rRNA gene sequencing. This is the first study to evaluate the changes in the gastric microbiota based on the reconstructive method employed.

## Methods

### Patients

From January 2019 to April 2020, 151 patients with gastric cancer underwent gastrectomy at the Osaka Medical and Pharmaceutical University Hospital, Takatsuki City, Japan. The following patients were excluded from the current analyses: 20 patients who underwent total gastrectomy, 15 who underwent proximal gastrectomy, 2 who underwent partial gastrectomy, 1 who underwent pylorus-preserving gastrectomy, and 8 who underwent remnant gastrectomy. We also excluded 2 patients who had macroscopic residual disease at the time of surgery (R2 resection), 4 who underwent neoadjuvant chemotherapy, 2 who underwent simultaneous resection of other organs for malignant diseases, 10 who had pyloric stenosis, and 16 who were on the following medications: immunosuppressive drugs (n = 1), corticosteroids (n = 2), and acid-suppressing drugs (n = 13). Hence, altogether 71 patients who underwent DG for gastric cancer were included, out of which none used antibiotics during the 3 months preceding surgery.

Lymph node dissection, surgical procedures, and gastric reconstruction were performed according to the Japanese Gastric Cancer Treatment Guidelines^11^. At our institute, BI reconstruction is the first-line reconstruction method after DG. BI reconstruction with delta-shaped mechanical anastomosis was performed^12^. In cases involving a small remnant stomach, hiatal hernia, or reflux esophagitis, RY reconstruction was performed^13^. Of the 71 patients who underwent DG, 40 underwent reconstruction using the BI method, and 31 using the RY method. The patients’ pathological stages were defined based on the Japanese classification of gastric carcinoma^14^.

This prospective cohort study was conducted in accordance with the Declaration of Helsinki and its latest amendments. The prospective cohort study was approved by the Osaka Medical and Pharmaceutical University Ethics Committee, Takatsuki City, Japan (approval no.: 2145; approval date: 8/5/2017). Written informed consent was obtained from all participants.

### Gastric sample collection

A preoperative endoscopic examination was performed in each case to evaluate and mark the tumor. Patients were advised to keep an overnight fast until the procedure on the following morning and no pre-procedural prophylactic antibiotics were prescribed. Simultaneously, gastric samples were obtained using a disposable syringe connected to the tube to aspirate as much gastric fluid as possible. After collection, the sample was immediately frozen at − 80 °C before deoxyribonucleic acid (DNA) extraction. Upper gastrointestinal endoscopic examination was performed 6 months after gastrectomy as part of the postoperative workup, and gastric samples were collected in the same manner. None of the patients were on acid-suppressing drugs post DG as removal of gastric fundic glands, the primary source of gastric acid, leads to lower acid concentrations in the remnant stomach.If the amount of gastric fluid was small, the samples were collected with sterile water evenly distributed in the stomach. Effluents from the gastroscope and diluent solutions and media were used as negative controls for bacterial growth to investigate potential contamination.

### DNA extraction, 16S rRNA sequencing, and taxonomic classification

DNA extraction, 16S rRNA sequencing, and taxonomic classification were performed using a slightly modified version of methods we have employed previously for saliva samples^15^. The collected gastric fluids were homogenized with 0.1 mm glass beads using a Disruptor Genie homogenizer (Scientific Industries Inc., Bohemia, NY, USA) for 90 s. Extraction of bacterial genomic DNA was performed from the homogenized samples using GENE PREP STAR PI-480 (Kurabo Industries Ltd.) following the manufacturer’s instructions. The V1–V2 region of the 16S rRNA gene was amplified for 25 cycles using the primer set 27 Fmod: 5′-TCGTCGGCAGCGTCAGATGTGTATAAGAGACA.

GAGRGTTTGATYMTGGCTCAG-3′ and 338R: 5′-GTCTCGTGGGCTCGGAGATG TGTAGTGTATAAGAGACAGTGCTGCCTCCCGTAGGAGT-3′.

Each library was prepared using the 16S rRNA sequencing library preparation protocol (Illumina, San Diego, CA, USA). DNA sequencing was performed for 500 cycles using the MiSeq Reagent Kit v2 (Illumina). An average of 39,966 sequence reads with 250 bp paired-ends were denoised, and quality reads were filtered using DADA2 in Quantitative Insights Into Microbial Ecology 2 (QIIME2) version 2020.2^16^. The quality filtering resulted in 4,539,853 sequences, with a mean of 31,971 sequences per sample (minimum: 14,581; maximum: 56,932). We decided to set the minimum depth cut-off for rarefaction at 10,000 and retained all samples for downstream analysis^17^. The processed sequences were clustered into operational taxonomic units (OTUs), which were defined based on a cut-off of 97% similarity.

### Statistical analysis

All statistical analyses were performed using JMP pro 15 (ver. 15, SAS Institute, Cary, NC, USA). For participant characteristics, categorical variables are presented using frequency counts; intergroup comparisons were analyzed using Fisher’s exact test or the Wilcoxon rank-sum test. Continuous variables were compared using Student’s *t*-test. A *p*-value of less than 0.05 was considered reflective of statistical significance.

Statistical analysis was performed using a slightly modified version of our previous methods^15^. The within-patient alpha diversity of bacterial communities was assessed by the Shannon’s index and the observed OTU index, and the Kruskal–Wallis test was used to compare between groups. The beta diversity between patients was assessed based on the Bray–Curtis dissimilarity, with unweighted and weighted UniFrac distance metrics^18^. Global differences in the microbiome structure in the UniFrac analysis were visualized using a principal coordinate analysis. The significance of compositional differences between groups was assessed using permutational multivariate analysis of variance (PERMANOVA). These analyses were conducted using the QIIME2 software^18^.

The linear discriminant analysis effect size (LEfSe) algorithm was used to detect genera that differed in abundance between the two groups. All analyses were performed with the α parameter of LEfSe set to 0.05 for pairwise tests and the threshold of the logarithmic score for linear discriminant analysis set at 3.0.

## Results

### Patients’ characteristics

The eligible patients who underwent DG comprised 40 who underwent BI reconstruction and 31 who underwent RY reconstruction. According to the reconstructive method, patient characteristics are listed in Table 1. The mean ages of the patients who underwent BI and RY reconstructions were 71.1 and 70.3 years, respectively; the male:female ratios were 23:17 and 21:10, respectively; the American Society of Anesthesiologists scores (1/2/3) were 7/29/4 and 1/26/4, respectively; and the percentages of adjuvant chemotherapy administered were 15% and 25.8%, respectively. There were no significant differences in background characteristics.

**Table 1:** Characteristics of patients with BI and RY reconstruction.

	Billroth I n = 40	Roux-en-Y n = 31	*P* value
Sex (M/F)	23/17	21/10	0.46
Age (years)	71.1 ± 8.7	70.3 ± 9.2	0.46
ASA score (1/2/3)	7/29/4	1/26/4	0.16
*Helicobacter pylori* detection	18 (45%)	12 (38.7%)	0.6
**Lymph node dissection**			
(DI/DI+/D2/D2+)	1/35/3/1	1/21/8/1	0.15
cStage (I/II/III)	37/2/1	21/4/5	0.07
**Surgical approach**			
(Laparoscopic/open/robotic)	36/2/2	24/5/2	0.27
Adjuvant chemotherapy (%)	7 (15%)	8 (25.8%)	0.6

*ASA* American Society of Anesthesiologists, *BI* Billroth I, *RY* Roux-en-Y.

### Microbiota composition before and after DG

**Figure 2:**
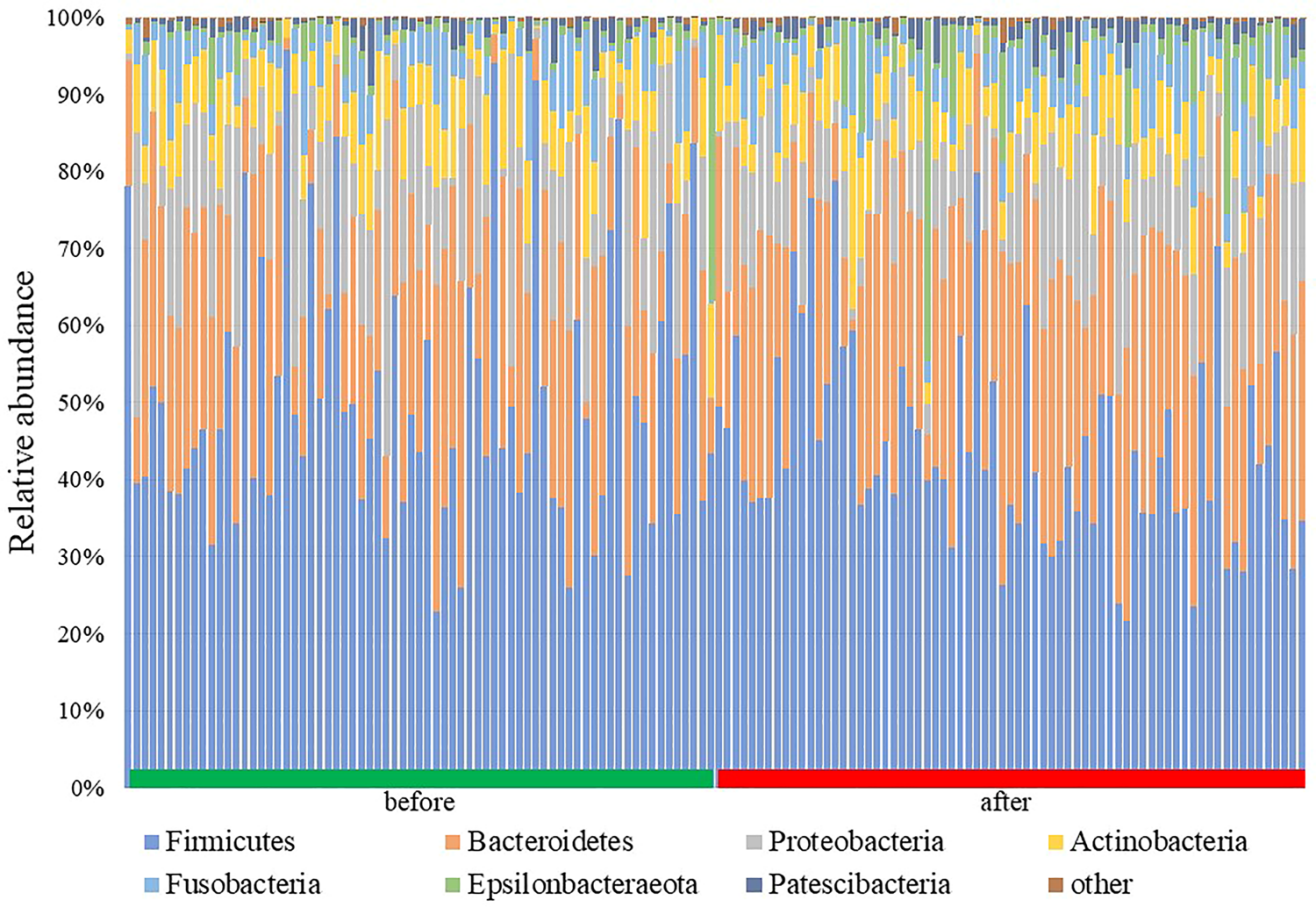
Taxonomic composition of the gastric microbiota before and after distal gastrectoy (DG) at the phylum level. The predominant bacteria (> 5% of the total sequences in either group) at the phylum level included Firmicutes, Bacteroidetes, Proteobacteria, and Actinobacteria, which comprised 91.4% and 88.3% of the gastric microbiota before and after DG, respectively.

Gastric bacteria with a relative abundance of at least 0.1% in the before and after DG groups were classified into 8 phyla, 13 classes, 29 orders, 31 families, and 50 genera. The predominant bacteria (> 5% of the total sequences in either group) at the phylum level included Firmicutes, Bacteroidetes, Proteobacteria, and Actinobacteria, which comprised 91.4% and 88.3% of the gastric microbiota before and after DG, respectively (Fig. 2).

**Figure 3:**
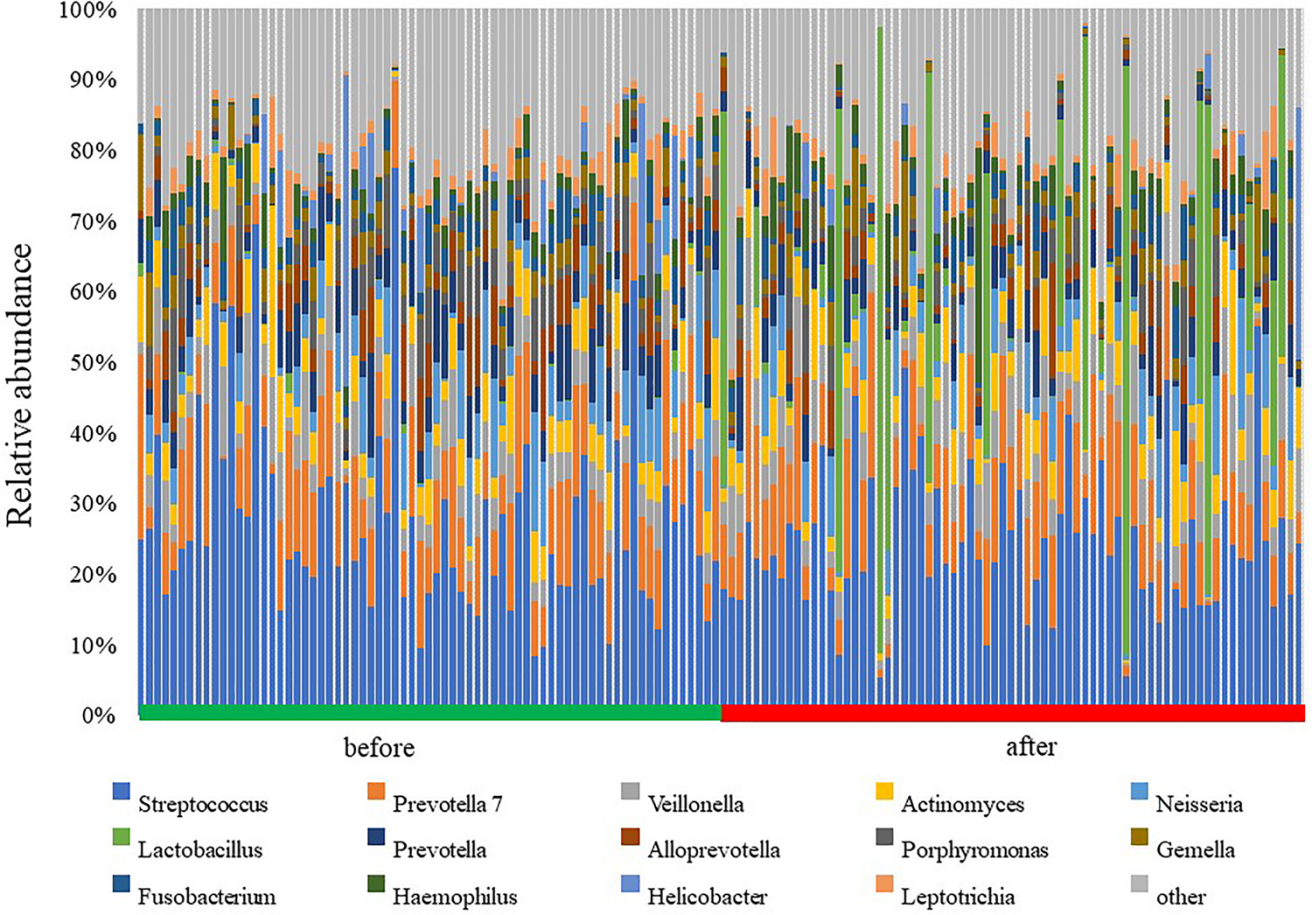
Taxonomic composition of gastric microbiota before and after distal gastrectomy (DG) at the genus level in Billroth I (BI) and Roux-en-Y (RY) reconstructions. Bar plot of the 14 most abundant genera in patients before and after DG. The most abundant genera before and after DG were *Streptococcus*, *Prevotella 7*, *Lactobacillus*, *Veillonella*, and *Actinomyces*.

Before DG, the genus level was characterized by six genera (*Streptococcus*, *Prevotella 7*, *Veillonella*, *Neisseria*, *Actinomyces*, and *Prevotella*), of which *Neisseria* and *Prevotella* were absent after DG. In contrast, the samples obtained after DG included five genera (*Streptococcus*, *Prevotella 7*, *Lactobacillus*, *Veillonella*, and *Actinomyces*), of which *Lactobacillus* was absent before DG (Fig. 3). The predominant bacteria at the genus level included *Streptococcus* with a mean relative abundance of 27.1% and 24.0%, and *Prevotella 7* with a mean relative abundance of 11.3% and 10.2% before and after DG, respectively.

### Helicobacter pylori infection

*H. pylori* is a microaerophilic, spiral-shaped, gram-negative bacteria belonging to the order Campylobacterales^19^ and is the cause of most gastric cancers^20^. Before DG, *H. pylori* was detected in the gastric fluid of 31 patients (43%), whereas after DG, *H. pylori* infection was identified in 20 patients (28%). Before DG, the percentages of patients with *H. pylori* in the BI and RY reconstruction groups were 47.5% and 41.9%, respectively, with no significant between-group differences (Table 1). Evidence for postoperative eradication of *H. pylori* and recommendations for the same in the Japanese Gastric Cancer Treatment Guidelines^11^ are lacking; therefore, it is not routinely undertaken.

### Microbial diversity of gastric fluid before and after DG

**Figure 4:**
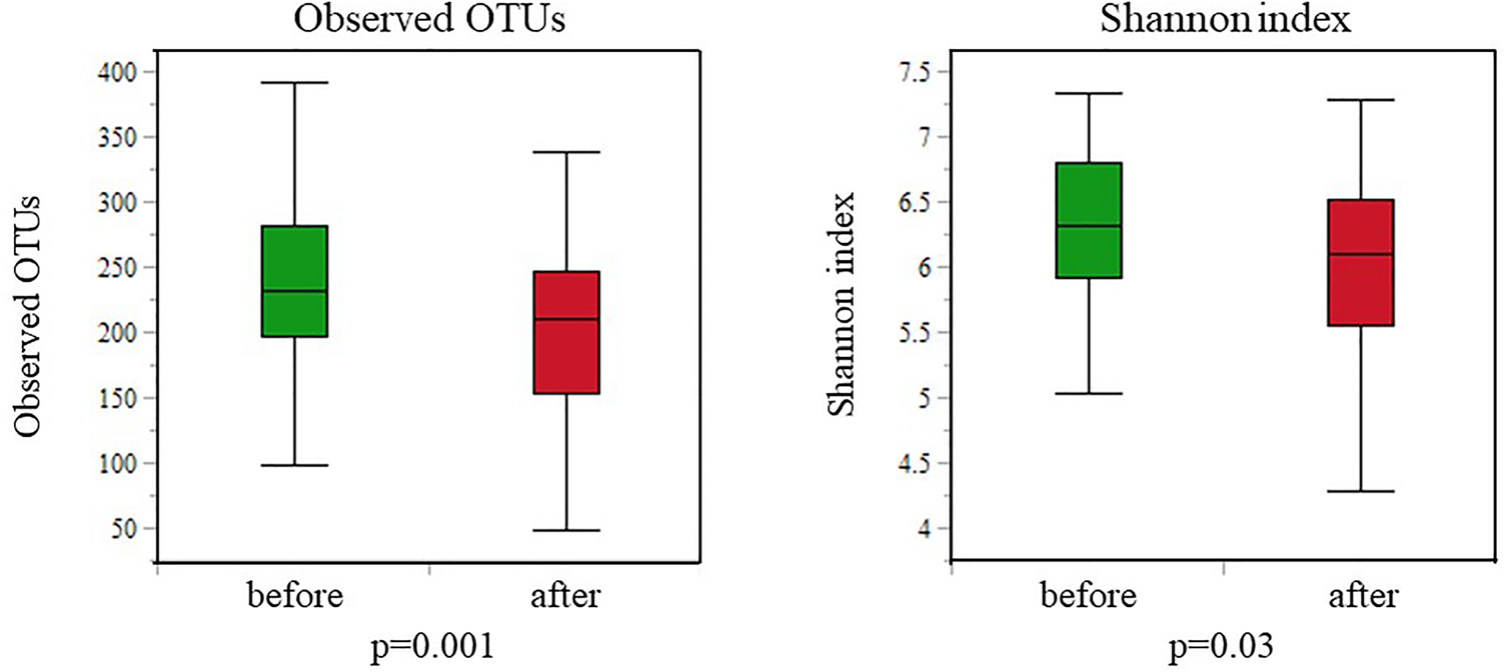
Alpha diversity of the gastric microbiota before and after distal gastrectomy (DG). Comparisons of the operational taxonomic unit and Shannon indices of the gastric microbiota before and after DG. P-values from the Kruskal–Wallis test are shown.

As shown in Fig. 4, the species richness (alpha diversity) was much lower in the gastric microbiota after than before DG (observed OTUs, *p* = 0.001; Shannon’s index, *p* = 0.03).

**Figure 5:**
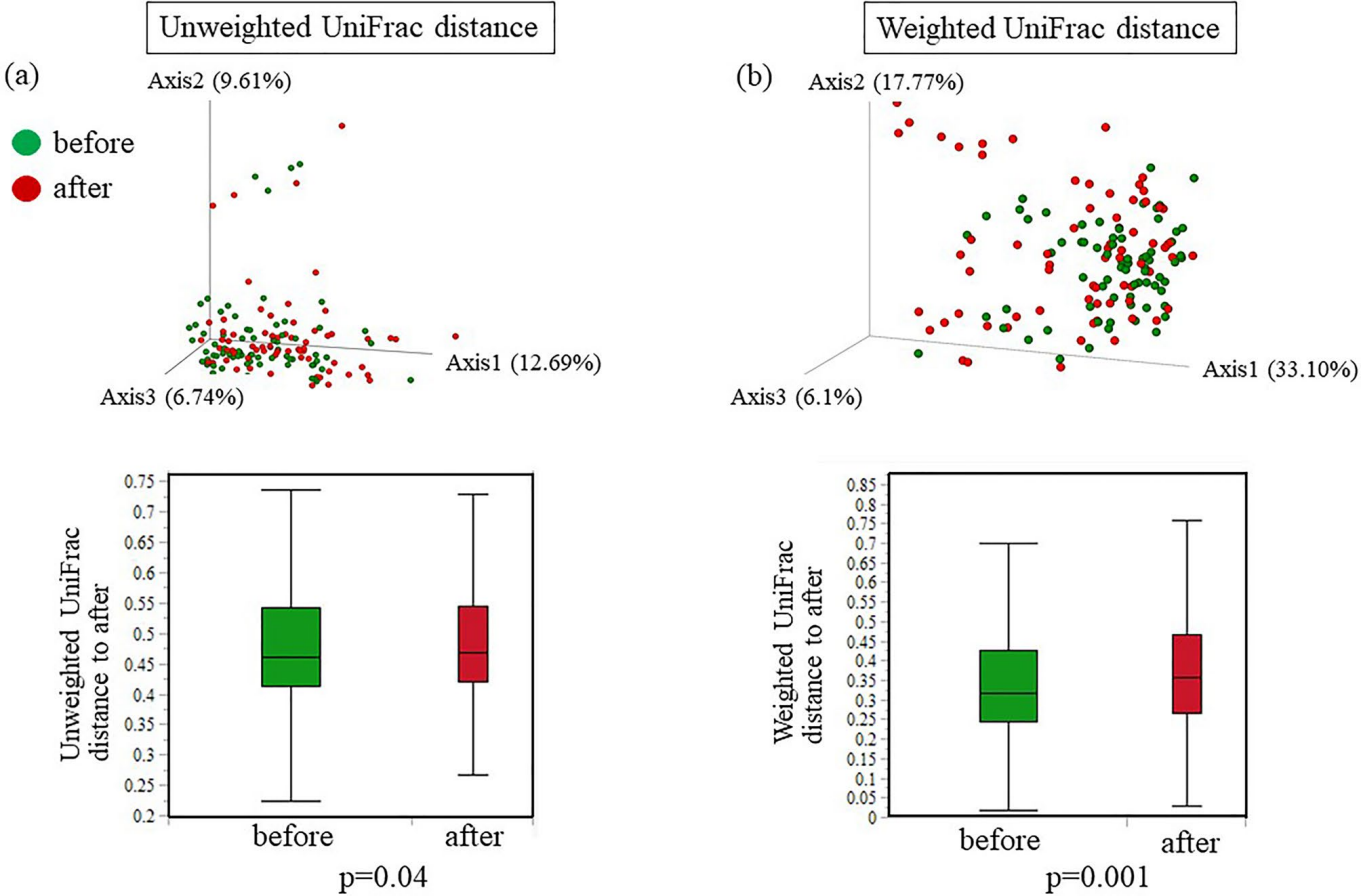
Beta diversity of the gastric microbiota before and after distal gastrectomy (DG). (**a**) Unweighted UniFrac distances. (**b**) Weighted UniFrac distances. Principal coordinate analysis plot for samples before and after DG. Box plots represent UniFrac distances of the before and after groups relative to the after group, *p* < 0.05; comparison between groups using a permutational multivariate analysis of variance (999 permutations).

The principal coordinate analysis of the unweighted and weighted UniFrac distance metric-based results revealed that the gastric samples differed between the “before DG” group and the “after DG” group in three-dimensional space (Fig. 5). This between-group compositional variation was verified by a PERMANOVA based on UniFrac data (unweighted, *p* = 0.04; weighted, *p* = 0.001). A PERMANOVA using Bray–Curtis metrics based on the relative abundance of bacterial genera also revealed a significant difference before and after DG *(p* = 0.001).

### Linear discriminant analysis effect size

**Figure 6:**
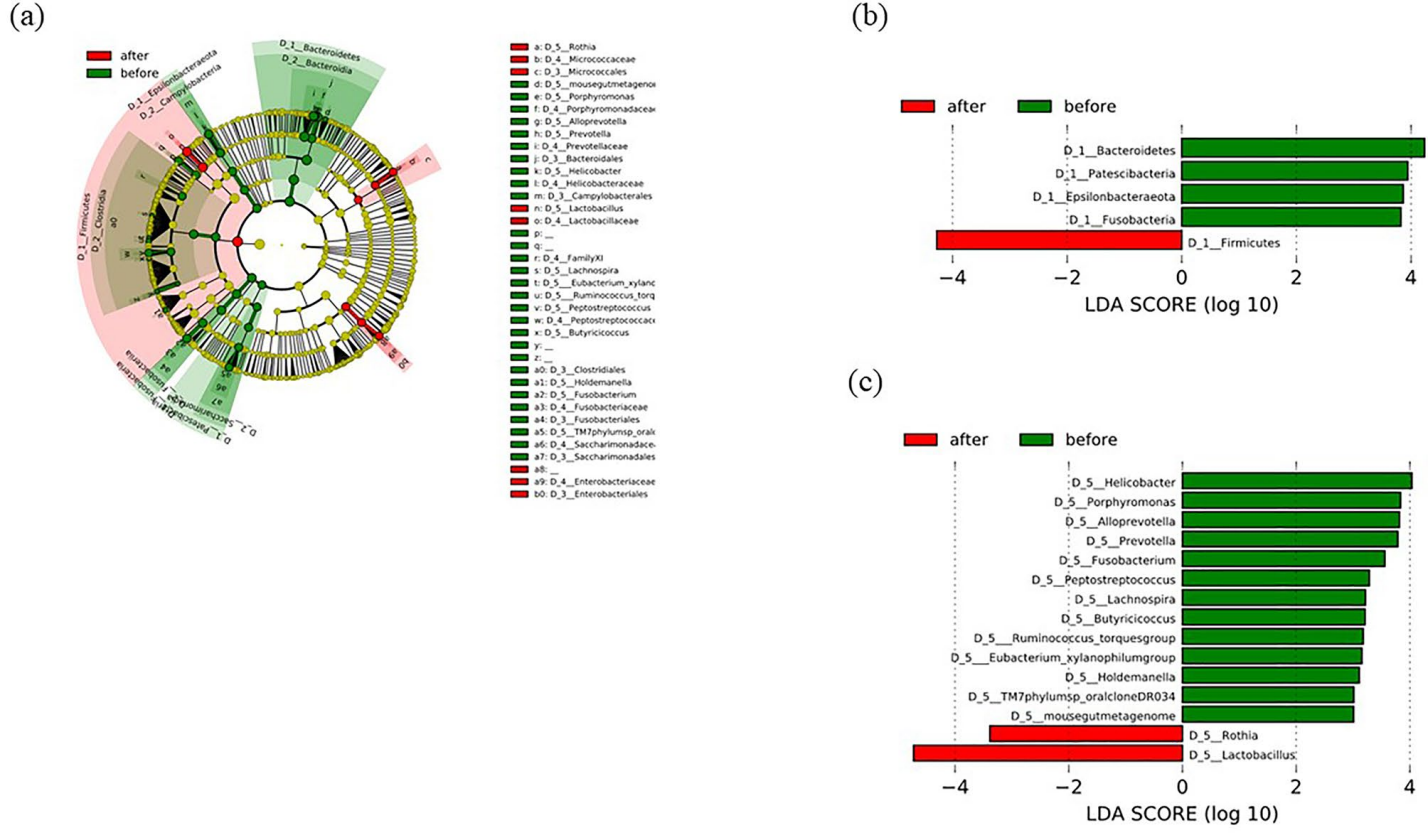
Differences in the abundance of bacterial genera before and after distal gastrectomy (DG) identified by linear discriminant analysis (LDA) effect size. (**a**) Cladogram of differentially abundant bacterial taxa in which each layer represents a different taxonomy. The enriched taxa of the gastric microbiome after DG are illustrated in the cladogram. The central point represents the tree’s root (Bacteria), and each ring represents the next lowest taxonomic level (phylum to genus: 1, phylum; 2, class; 3, order; 4, family; 5, genus). Each circle’s diameter represents the relative abundance of taxa. (**b**) LDA scores of phylum levels. (**c**) LDA score at the genus level. Histogram of LDA scores for differentially abundant bacterial taxa before and after DG. LDA score ≥ 3.0 is shown. Red represents significantly abundant taxa before DG compared to those after DG. Green represents the most abundant taxa after DG.

The cladogram in Fig. 6a represents the significantly different taxa before and after DG, with a hierarchy reflecting the taxonomic rank from phylum to genus. We observed that before DG, there was a significantly higher abundance of Bacteroidetes, Epsilonbacteraeota, Fusobacteria, and Patescibacteria, and a lower abundance of Firmicutes (Fig. 6b). Regarding genera, LEfSe revealed an increased abundance of *Helicobacter*, *Porphyromonas*, *Alloprevotella*, *Prevotella*, *Fusobacterium*, *Peptostreptococcus*, *Holdemanella*, and *Mousegutmetagenoma* and a reduction in *Rothia* and *Lactobacillus* before DG compared with those after DG (Fig. 6c).

### Microbial diversity of gastric fluid in BI reconstruction and RY reconstruction after DG

**Figure 7:**
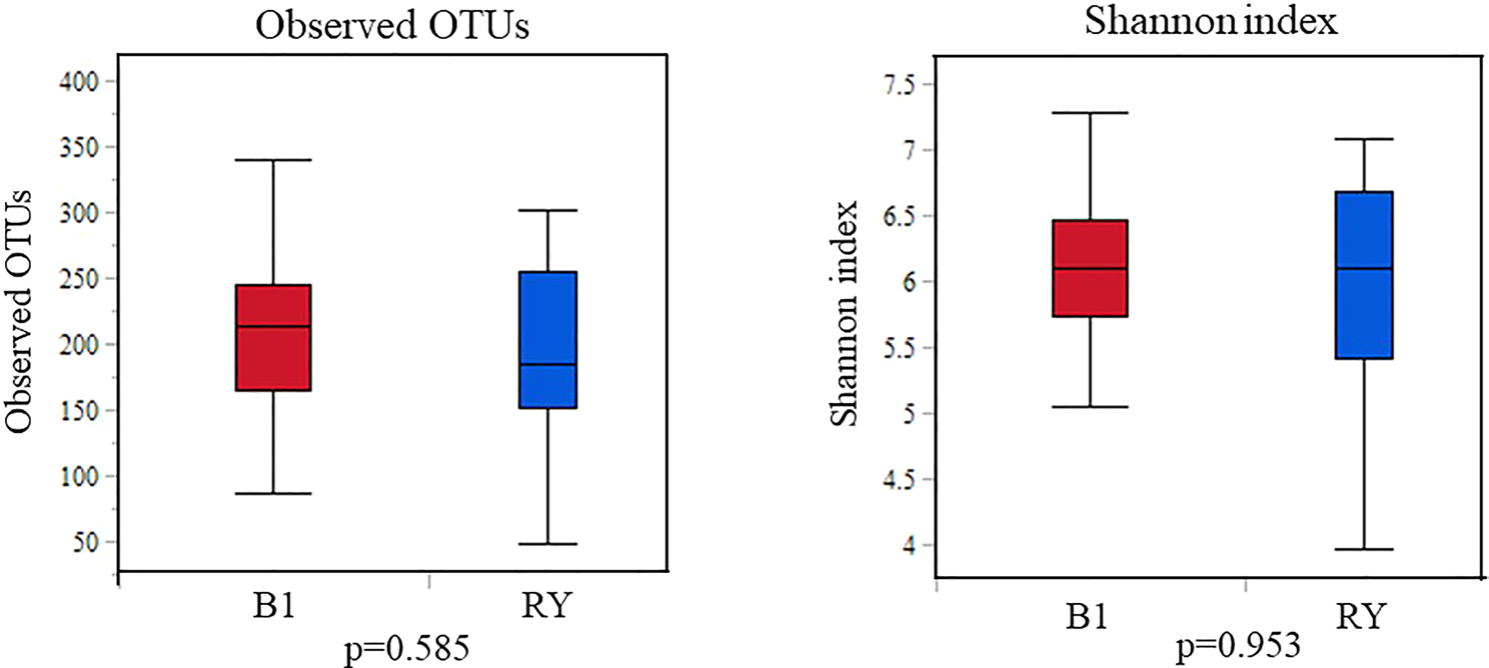
Alpha diversity of the gastric microbiota between Billroth I (BI) and Roux-en-Y (RY) reconstruction. Operational taxonomic unit and Shannon indices of the gastric microbiota after BI compared with RY reconstruction. P-values from the Kruskal–Wallis test are shown.

**Figure 8:**
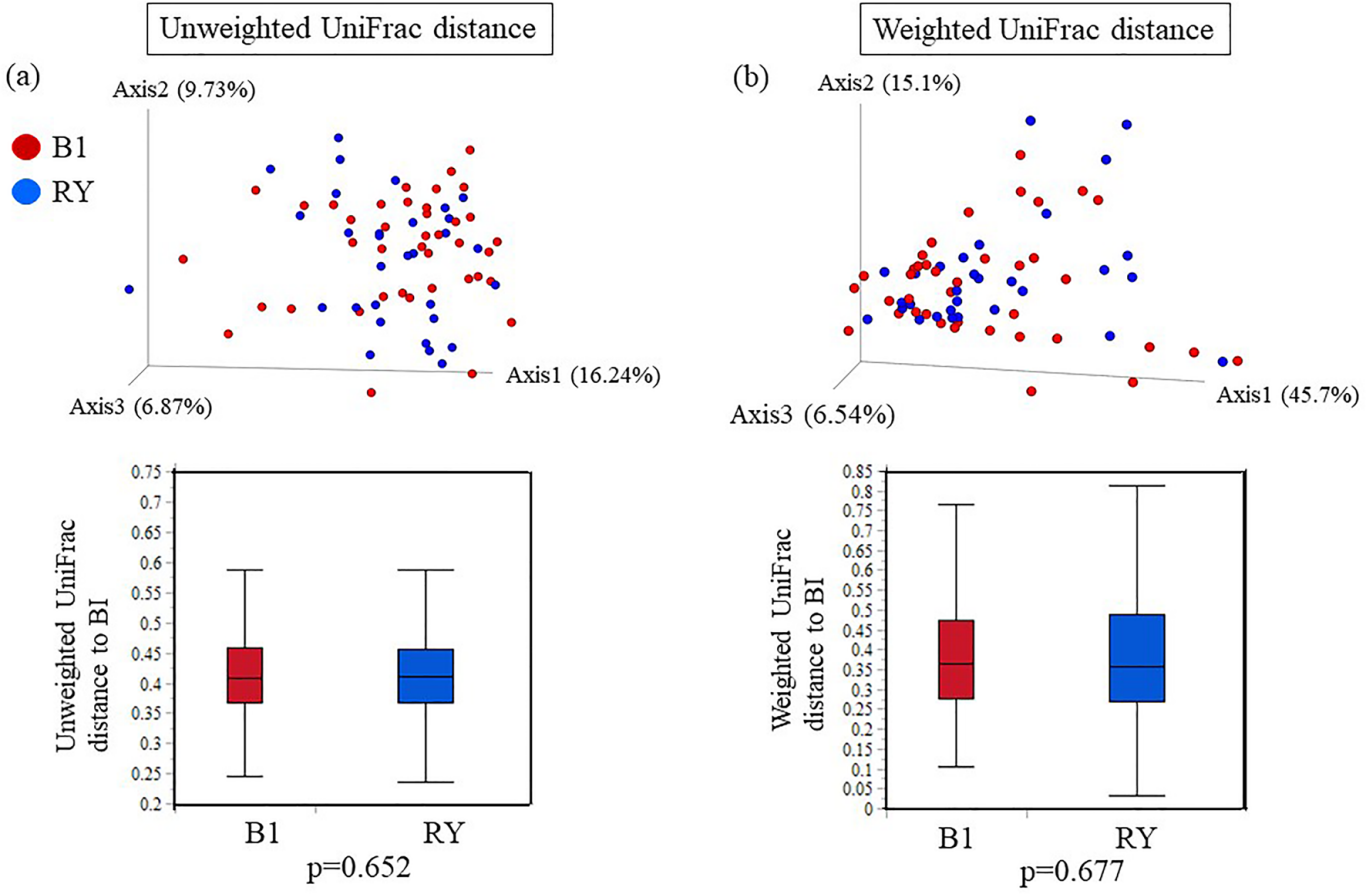
Beta diversity of the gastric microbiota between Billroth I (BI) and Roux-en-Y (RY) reconstruction. (**a**) Unweighted UniFrac distances. (**b**) Weighted UniFrac distances. Principal coordinate analysis plot for BI and RY reconstruction samples. Box plots represent UniFrac distances for RY reconstruction from BI reconstruction. *p* < 0.05, comparison between groups using a permutational multivariate analysis of variance (999 permutations).

As shown in Fig. 7, there were no statistically significant differences in the gastric microbiota after DG in the BI and RY reconstruction groups regarding alpha diversity (observed OTUs, *p* = 0.58; Shannon’s index, *p* = 0.95). Further, beta diversity was not significantly different between the two groups (unweighted, *p* = 0.65; weighted, *p* = 0.67; Bray–Curtis, *p* = 0.63) (Fig. 8). Moreover, LEfSe analysis did not detect any bacterial genera with significantly different abundances between the two groups.

## Discussion

This study examined the gastric microbiota prepared from the gastric fluid of patients who underwent either BI or RY reconstruction after DG for gastric cancer, using 16S rRNA sequencing analysis. To our knowledge, this is the first report to compare the composition of gastric microbiota prepared from gastric fluid in BI and RY reconstructions. We have research experience with gastric fluid on patients with gastric cancer^21^. Our results indicate a change in the gastric microbiota before and after gastric resection; interestingly, no significant differences were observed between the two different reconstructive methods in terms of diversity and community composition.

The alpha diversity of the gastric microbiota after DG was significantly lower than that before DG. After DG, the gastric environment changes dramatically; for example, there is reduced gastric acid secretion due to a reduction in the fundic gland region and bile regurgitation. In contrast to our results, Tseng et al. reported that in six cases (deemed to be a relatively small sample size) with Billroth II reconstruction, the gastric microbiota was significantly more diverse after DG^7^. They employed a different methodology from that utilized in this study, in that the sample was retrieved from the gastric mucosa and sample collection was performed 2 years after DG.

It has been recently reported that the diversity and composition of the gastric microbiota differ significantly according to the location: whether around the tumor, peritumor, or in the normal region of the stomach^22^. It was thought that after stomach resection for gastric cancer, whereby the tumor and peritumor area would have been resected, then the remnant stomach would naturally comprise only the normal region. Therefore, only the gastric microbiota of the original normal region would be retained in the microbiota present in the remnant stomach. However, our technique of collecting the gastric fluid can represent the inclusive microenvironment of the stomach.

We compared the gastric microbiota after DG with and without postoperative adjuvant chemotherapy. Since there was no significant difference in both alpha and beta diversity (Supplementary Figs. S1, S2), we judged that the effect of postoperative adjuvant chemotherapy on the gastric microbiota was small and included cases with postoperative adjuvant chemotherapy in our study.

In our investigation, the genera *Helicobacter*, *Prevotella*, *Fusobacterium*, and *Peptostreptococcus* were significantly reduced after DG, and these genera were reported to be relatively abundant in the tumor and peritumor regions of the stomach^22^.

The beta diversity exhibited a significant difference before and after DG. As a result of changes in the gastric environment due to the altered gastrointestinal tract, there is an apparent shift in the community composition of the gastric microbiota after DG. Therefore, the gastric microbiota differed before and after surgery. This result is consistent with those of a previous report^7^. The beta diversity of the gastric microbiota significantly increased with the use of acid-suppressing agents, including proton-pump inhibitors^23^. This situation is considered relatively similar to the environment after DG due to reduced gastric acid secretion. Therefore, it is in agreement with our beta diversity results.

In our study, the microbiota of the remnant stomach was dominated by four phyla: Proteobacteria, Firmicutes, Bacteroidetes, and Actinobacteria (Fig. 2), with a significantly higher abundance of Firmicutes (Fig. 6b). Tseng et al. also reported that the stomach was dominated by the same four phyla after DG, with a significantly higher abundance of Firmicutes^7^. Our LEfSe analyses revealed that the proportions of genera *Rothia* and *Lactobacillus* were significantly higher after DG (Fig. 6c). Both genera, *Rothia* and *Lactobacillus*, are oral commensals, and it is believed that the oral microbiota moves into the remnant stomach and coexists.

A long-term (> 8 years) follow-up study reported an increased richness and diversity of the gut microbiota on stool sample analysis after subtotal gastrectomy with RY reconstruction^24^. A decrease in gastric acid secretion promotes the growth of *E. coli* and the migration of the oral microbiota through the remnant stomach to the large intestine is considered to affect the lower gastrointestinal microbiota. Over time, the upper gastrointestinal microbiota including gastric microbiota may increase in richness, as well as the lower gastrointestinal microbiota.

No significant differences in diversity and community composition between BI and RY reconstructions were observed in this study. Many reports have compared BI and RY reconstruction after DG in terms of quality of life and dysfunction^25–34^. The incidence of remnant gastritis and bile regurgitation is higher after BI than after RY reconstruction^25,26,28,29,31–33^; the procedures are generally equivalent regarding postoperative quality of life^27,28,31,32,34^.

It has been established that there is a discrepancy between endoscopic findings and a patient’s symptoms. The gastric microbiota of patients with functional dyspepsia is altered compared to that in healthy controls^35^. Alterations in the gastric microbiota are thought to cause postprandial distress and epigastric pain syndrome. The lack of a difference in the bacterial microbiota between BI and RY reconstruction in this study means that the gastric environment was comparable, possibly suggesting that both reconstructive methods were generally equivalent in terms of postoperative quality of life. However, since there are few published reports in this field and much remains unknown, further research on this topic is needed. Furthermore, examining the relationship between the quality of life and gastric microbiota may provide newer strategies for the betterment of the postoperative quality of life and has important clinical implications.

The present study has several limitations. First, we aspirated gastric fluid and examined the gastric microbiota, but the amount of gastric fluid that could be aspirated was limited, which could have caused individual differences. Although some reports collected gastric mucosal tissue, the gastric mucosal microbiota differs based on the collection site (e.g., the normal, peritumor, and tumor sites)^6,7,22^. Gastric fluid is spread evenly throughout the stomach. In this respect, gastric fluid aspiration has the advantage of evenly reflecting the gastric environment. In addition to the difference in the collected samples between this study and others, the measurement times are different. It is unknown when the bacterial microbiota changes; therefore, following the alterations in the bacterial microbiota over time is necessary. Knowledge of these changes and how they are affected by fluctuations in the gastric environment is of importance because it may potentially hold the keys to improved treatment approaches. Finally, in approximately half the patients, *H. pylori* was detected in the gastric fluid and was not unified. However, the percentage of *H. pylori* in the gastric fluid after DG is extremely low; hence, we believe that it has little effect on the comparison of gastric microflora with stomach microflora based on the method of reconstruction.

## Conclusion

We investigated the composition and diversity of gastric microbiota before and after DG. Although there were significant differences before and after DG, there were none in terms of diversity, composition, and community between BI and RY reconstructions.

## Supplementary Information

Supplementary Information.

## Data Availability

The data and code can be freely accessed at https://www.ncbi.nlm.nih.gov/bioproject/PRJDB12280 and used by researchers worldwide. They can also be requested from the corresponding author.
