# Supplementary material for: Comparison of the gastric microbiome in Billroth I and Roux-en-Y reconstructions after distal gastrectomy
Source: Sci Rep. 2022 Jun 22;12:10594. doi: 10.1038/s41598-022-14886-4 (PMC9217802; doi:10.1038/s41598-022-14886-4)
Supplement: Supplementary file 1 — Supplementary Information. [file 41598_2022_14886_MOESM1_ESM.docx]

**
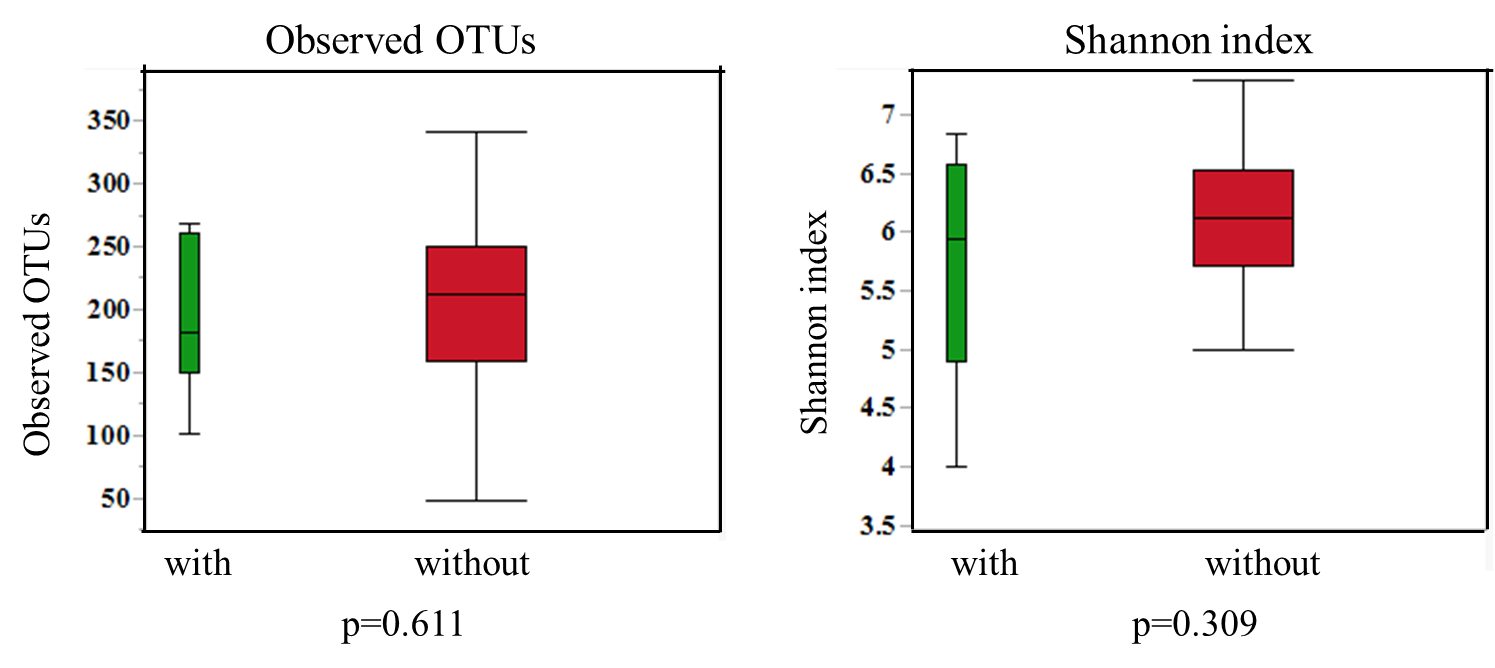
**

**Supplementary Figure 1: Alpha diversity of the gastric microbiota with and without postoperative adjuvant chemotherapy after DG**. Comparisons of the operational taxonomic unit and Shannon indices of the gastric microbiota with and without postoperative adjuvant chemotherapy after DG.

P-values from the Kruskal–Wallis test are shown.


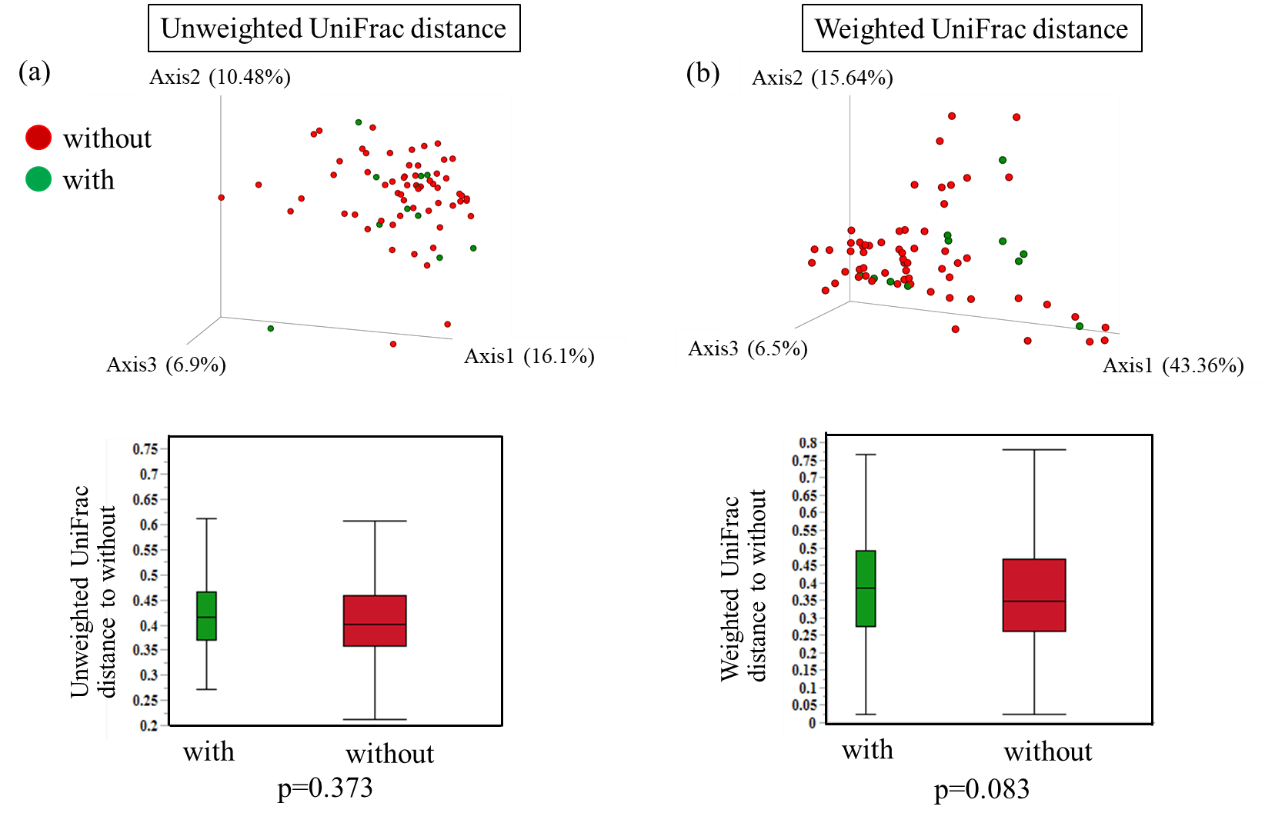


**Supplementary Figure 2: Beta diversity of the gastric microbiota with and without postoperative adjuvant chemotherapy after DG.** (a) Unweighted UniFrac distances. (b) Weighted UniFrac distances.

Principal coordinate analysis plot for samples with and without postoperative adjuvant chemotherapy after DG.

Box plots represent UniFrac distances of the with and without groups relative to that in the without group, P<0.05; comparison between groups using a permutational multivariate analysis of variance (999 permutations).
